# Natural language processing for disease phenotyping in UK primary care records for research: a pilot study in myocardial infarction and death

**DOI:** 10.1186/s13326-019-0214-4

**Published:** 2019-11-12

**Authors:** Anoop D. Shah, Emily Bailey, Tim Williams, Spiros Denaxas, Richard Dobson, Harry Hemingway

**Affiliations:** 1 Health Data Research UK London, University College London, 222 Euston Road, London, NW1 2DA UK; 2 Institute of Health Informatics, University College London, 222 Euston Road, London, NW1 2DA UK; 3 The National Institute for Health Research University College London Hospitals Biomedical Research Centre, University College London, 222 Euston Road, London, NW1 2DA UK; 4 University College London Hospitals NHS Foundation Trust, 235 Euston Road, London, NW1 2BU UK; 5 Clinical Practice Research Datalink, Medicines and Healthcare products Regulatory Agency, 10 South Colonnade, London, E14 4PU UK; 6 Department of Biostatistics and Health Informatics, King’s College London, De Crespigny Park, Denmark Hill, London, SE5 8AF UK

**Keywords:** Free text, Myocardial infarction, Primary care, Chest pain, Natural language processing

## Abstract

**Background:**

Free text in electronic health records (EHR) may contain additional phenotypic information beyond structured (coded) information. For major health events – heart attack and death – there is a lack of studies evaluating the extent to which free text in the primary care record might add information. Our objectives were to describe the contribution of free text in primary care to the recording of information about myocardial infarction (MI), including subtype, left ventricular function, laboratory results and symptoms; and recording of cause of death. We used the CALIBER EHR research platform which contains primary care data from the Clinical Practice Research Datalink (CPRD) linked to hospital admission data, the MINAP registry of acute coronary syndromes and the death registry. In CALIBER we randomly selected 2000 patients with MI and 1800 deaths. We implemented a rule-based natural language engine, the Freetext Matching Algorithm, on site at CPRD to analyse free text in the primary care record without raw data being released to researchers. We analysed text recorded within 90 days before or 90 days after the MI, and on or after the date of death.

**Results:**

We extracted 10,927 diagnoses, 3658 test results, 3313 statements of negation, and 850 suspected diagnoses from the myocardial infarction patients. Inclusion of free text increased the recorded proportion of patients with chest pain in the week prior to MI from 19 to 27%, and differentiated between MI subtypes in a quarter more patients than structured data alone. Cause of death was incompletely recorded in primary care; in 36% the cause was in coded data and in 21% it was in free text. Only 47% of patients had exactly the same cause of death in primary care and the death registry, but this did not differ between coded and free text causes of death.

**Conclusions:**

Among patients who suffer MI or die, unstructured free text in primary care records contains much information that is potentially useful for research such as symptoms, investigation results and specific diagnoses. Access to large scale unstructured data in electronic health records (millions of patients) might yield important insights.

## Background

**Fig. 1:**
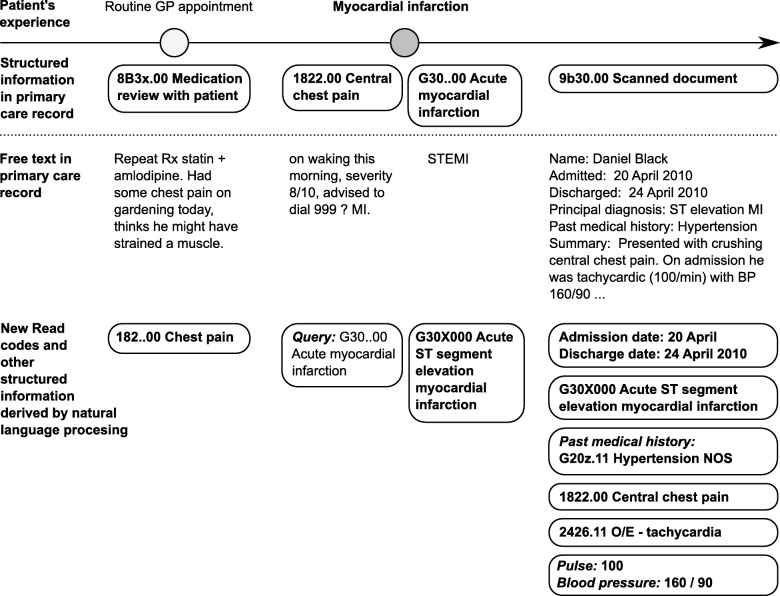
Illustration of patient’s experience, information entered in the structured part of the primary care record (Read codes), and additional information that might be available in free text. In this hypothetical example, the subtype of myocardial infarction and preceding symptoms are present only in the free text

Electronic health records (EHR) are increasingly used for clinical research, but much of the information they contain is stored in an unstructured way [1, 2]. Research projects using EHR databases conventionally use only the structured information, but could potentially miss important information if it is not coded correctly (Fig. 1). There has been increasing interest in using natural language processing (NLP) to extract additional information from the free text for research, such as in the eMERGE hospital network in the US [3]. However, there have been few studies using NLP on primary care data, which is crucial for understanding early manifestations of disease (before a patient is admitted to hospital or attends a secondary care clinic). This may enable the development of early diagnosis and treatment strategies.

For example, a previous study using structured information in primary care data found more than a 5-fold increase in the frequency of chest pain consultations in the two months prior to a myocardial infarction (MI) [4]. If some consultations for chest pain are not recorded using appropriate codes, as suggested in US studies [5], the prevalence of chest pain prior to MI may be underestimated. Accurate information on such symptoms is essential to inform public health endeavours aimed at preventing MI, but has not previously been studied on a large scale in the UK.

We used primary care data from the Clinical Practice Research Datalink (CPRD), a population-based source of longitudinal clinical information. Although early studies using CPRD manually reviewed small samples of text to validate coded diagnoses [6], there has been little research on the potential contribution of free text beyond the coded information in UK primary care, and previous studies have been limited to a few hundred texts [7–10].

At the time of this study, free text from CPRD primary care was stored at the Department of Health, and could be released to researchers after manual anonymisation by CPRD staff. This was time-consuming and costly, feasible for only small samples of text for validation studies. However, free text is no longer collected or made available by CPRD because of confidentiality concerns, so it is essential to know what is missing from the structured data.

To address these challenges, we developed and validated a natural language processing system, the Freetext Matching Algorithm (FMA) [7]. This is an entity linking program which maps sequences of words to Read codes, using manually defined synonyms and patterns to recognise the context of words in the text. As well as diagnoses, it can extract symptoms, dates and laboratory test results. The output of the algorithm is structured, containing only codes and numeric values. In this paper we describe a pilot project to use FMA to analyse primary care free text at source, without needing manual anonymisation, thus analysing larger samples of text than has previously been possible.

Our specific aims were to describe the contribution of free text to the recording of information about MI in CPRD primary care data, including MI subtype, left ventricular function, laboratory results, symptoms, and whether the MI record related to a new or historic event. We also used FMA to investigate cause of death recording in general practice, comparing the results to the cause of death recorded in the death registry.

## Methods

### Study data source

We used linked electronic health records from four data sources in England (the CALIBER resource [11]), which contains primary care data from CPRD linked to administrative hospital records (Hospital Episode Statistics, HES) and death registrations from the Office for National Statistics (ONS). The CALIBER programme involved additional linkage with the Myocardial Ischaemia National Audit Project (MINAP), facilitated by CPRD, and contained data from 244 practices in England. We previously carried out a study of the completeness and diagnostic validity of MI records in CALIBER [12], which included 21,482 patients with a first MI recorded in either MINAP, HES, ONS or CPRD primary care in 2003–2009. For this pilot project we chose a random subset of 2000 patients from this study. This sample size would yield enough free text to demonstrate the value of this approach as it would be too large to anonymise manually, but it would be feasible to extract and analyse as a pilot project. We also studied 1800 patients who died of any cause between 2001 and 2009 and had a death registry record in linked ONS data (200 patients per year).

For the MI population, we analysed free text in the primary care record associated with clinical, test or referral events up to 90 days before or after the MI, and for the death population, we analysed free text in primary care associated with clinical or referral events on or after the date of death.

### Natural language processing

The Freetext Matching Algorithm (FMA) is a natural language processing system designed to extract Read codes and other structured data from UK general practice records. It was developed using small samples of pre-anonymised text from CPRD and is available under an open source license (GPL Version 3).

FMA has been described previously [7]; briefly it is a rule-based annotation and information extraction engine. The text is first cleaned of semi-structured computer-generated phrases (defined in a manual lookup), then converted to lower case and split into individual words. The program identifies dates, numbers and words, and maps individual words to lookup tables of ‘medical’ words (any word contained within any Read term) and ‘non-medical’ words (from an English lexicon). If a word does not match any entry in the dictionaries, it is assumed to be misspelt, and the program attempts spelling correction with a single letter insertion or substitution algorithm. Attributes such as negation are identified by sequential application of regular expression rules, and the program then attempts to match sequences of up to five words to Read terms. If the text phrase does not match a Read term exactly, parts of the phrase are substituted by alternative words and phrases using the synonym table. A custom scoring function rates the quality of each potential match, and returns the Read term with the closest match above a minimum threshold. The output of the algorithm is a sequence of Read terms or quantitative data with attributes.

We tested the FMA on pre-anonymised samples of free text from patients with coronary artery disease, and added terms to the lookup tables to enable it to detect subtype of myocardial infarction and left ventricular function. We collaborated with CPRD to arrange for their staff to run the program on free text for the study population. CPRD staff verified that the output contained only coded or numeric data before releasing it to researchers. We used FMA in preference to other open source NLP tools because it was small (a single 200 KB executable and 8.5 MB lookup tables) and required no installation or special software, so it was easy for CPRD staff to run. It also had the advantage of being customised for text in primary care records, returning results in a similar format to existing structured CPRD data.

### Information extracted from free text

We summarised the frequencies of Read codes extracted by FMA with different data types within 90 days of myocardial infarction. We calculated the frequencies of recording in Read codes and free text for symptoms and investigation results of particular interest, such as chest pain, shortness of breath, pulse rate, angiogram results and left ventricular function.

### Classifying the type of myocardial infarction

For patients who had a MINAP record in CALIBER, i.e. those who arrived at hospital alive and whose data were submitted to the national acute coronary syndrome registry, we investigated the accuracy of structured and unstructured information in the primary care record. We classified the type of myocardial infarction according to the closest STEMI or NSTEMI Read code or free text record after the date of MI within 30 days. We calculated the sensitivity and specificity of Read codes and free text for identifying the type of MI.

### Validity of myocardial infarction records

Two clinicians manually reviewed the CALIBER record (CPRD structured primary care record, linked data from HES, and information extracted by FMA), for patients with MI recorded in primary care but not HES or MINAP. They adjudicated whether or not the MI Read code in CPRD represented a true current MI, resolving any disagreements by discussion to reach a consensus. For patients with MI recorded in HES, MINAP or ONS within 30 days of the primary care record, we assumed that the MI was genuine and did not review their record manually.

We tested a machine learning algorithm (Random Forest [13]) on the task of discriminating between correct and incorrect MI records, using the manual adjudication or presence of a HES or MINAP record as the ‘gold standard’ for a true MI. Predictor variables for this task included the likelihood of the exact Read code to be associated with a HES or MINAP record for MI in other patients (Supplementary Table 7 in Herrett et al. [12]), specific details about the MI record (the consultation type, whether it was entered on the date it occurred, whether there was a coronary register entry on the same date, whether it was recorded as a ‘new’ or ‘continuing’ episode), whether there was a Read code for hospital admission within 7 days, and whether there was a Read code for chest pain or shortness of breath within 7 days. We also generated binary variables for the presence of the most common 100 Read codes entered on the same date, or dated within 30 days of the MI date, or Read codes extracted by FMA within 30 days of the MI date. We generated composite variables grouping Read codes by their first 1, 2 or 3 characters, in case groups of Read codes were better predictors than sparse variables encoding the presence of individual Read codes.

We used Random Forest with 100 trees, trying all variables at every split (to avoid bias due to a large proportion of sparse or non-informative variables). We calculated the accuracy of models generated from 200 bootstrap samples of the data, using the patients not selected as the test set for each model.

### Cause of death

We manually reviewed structured death certificate information, Read codes and diagnoses extracted by FMA from CPRD primary care data to assign the most likely underlying cause of death for the sample of 1800 patients. We converted the causes of death to ICD-10 using the Read to ICD-10 mapping table, and allocated the underlying cause of death by manual review of the extracted coded diagnoses and application of the ICD-10 selection rules [14], blinded to the cause of death recorded in the death registry (we did not have access to review the raw free text diagnoses). We calculated the proportion of deaths with cause recorded in different ways, giving priority to the more specific information (e.g. a free text diagnosis with death certificate category was given priority over a Read coded diagnosis without category). We compared the causes of death thus extracted with the gold standard cause in the death registry. We assessed the similarity of the underlying ICD-10 code and concordance for three common diagnosis groups: coronary heart disease, cerebrovascular disease and cancers.

### Statistical analysis

All statistical analysis was carried out using R Version 3.4 [15], using the packages CALIBERdatamanage and CALIBERcodelists (published on R-Forge [16]) to assist with data management.

## Results

FMA analysed 31,913 text entries in CPRD containing 705,523 words in 40 min on a Windows 2008 Server.

### Information extracted from free text for myocardial infarction patients

**Fig. 2:**
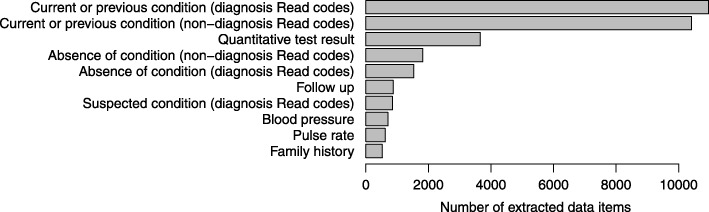
Data items extracted from primary care free text for 2000 patients within 90 days before or after myocardial infarction

We included 2000 MI patients in this study, with median age 75 years (interquartile range 63, 83), of whom 781 (39%) were female. FMA extracted 21,369 Read codes with the attribute ‘medical history’ or ‘current or previous condition’, of which 10,957 were diagnoses (defined as Read codes in the diagnosis chapter, rather than administration, procedures, test results etc.), and 1117 suspected conditions, of which 850 were diagnoses. FMA also extracted 3658 test results, 3313 statements of negation and 968 entries referring to hospital admission (Fig. 2). The most common negated conditions were chest pain (377 entries), breathlessness or dyspnoea (300), unspecified pain (208) and oedema (121). The most common Read coded diagnosis in the free text was ‘acute myocardial infarction’ (8.1%) (Table 1).

**Table 1:** Most common Read codes extracted from free text for 2000 patients within 90 days of MI in CALIBER (top five codes in each category)

Number of records (%)	Read code	Read term
Current or previous condition (diagnosis Read codes)		
887 (8.1%)	G30z.00	Acute myocardial infarction NOS
443 (4.1%)	R065.00	[D] Chest pain
304 (2.8%)	C10..00	Diabetes mellitus
272 (2.5%)	G307100	Acute non-ST segment elevation myocardial infarction
256 (2.3%)	G33..00	Angina pectoris
Current or previous condition (non − diagnosis Read codes)		
991 (9.5%)	8H3Z.00	Other hospital admission NOS
913 (8.8%)	8H…00	Referral for further care
795 (7.6%)	1M…00	Pain
469 (4.5%)	173..00	Breathlessness
445 (4.3%)	8HA..11	Discharged from follow up
Quantitative test result		
577 (15.8%)	42Z7.00	Red blood cell distribution width
142 (3.9%)	42M..00	Lymphocyte count
141 (3.9%)	42N..00	Monocyte count
139 (3.8%)	42K..00	Eosinophil count
138 (3.8%)	42J..00	Neutrophil count
Absence of condition (diagnosis Read codes)		
121 (7.8%)	R023.00	[D] Oedema
105 (6.8%)	G33..00	Angina pectoris
90 (5.8%)	R065.00	[D] Chest pain
57 (3.7%)	A....00	Infectious and parasitic diseases
38 (2.5%)	R006200	[D] Fever NOS
Absence of condition (non-diagnosis Read codes)		
281 (15.6%)	182..00	Chest pain
274 (15.2%)	173..00	Breathlessness
208 (11.5%)	1M…00	Pain
89 (4.9%)	2I18.12	O/E - tenderness
48 (2.7%)	199..00	Vomiting
Suspected condition (diagnosis Read codes)		
70 (8.2%)	G30z.00	Acute myocardial infarction NOS
48 (5.6%)	K190.00	Urinary tract infection, site not specified
40 (4.7%)	A….00	Infectious and parasitic diseases
32 (3.8%)	G33..00	Angina pectoris
23 (2.7%)	G581.13	Impaired left ventricular function
Suspected condition (non-diagnosis Read codes)		
44 (16.5%)	8H…00	Referral for further care
18 (6.7%)	8H3Z.00	Other hospital admission NOS
16 (6.0%)	1M…00	Pain
9 (3.4%)	173..00	Breathlessness
7 (2.6%)	2C2..11	O/E - anaemic

Among patients with MI not recorded in CPRD structured data, 108 (18.9%) had MI recorded in free text within 30 days. The proportion of patients with recording of symptoms and investigations associated with MI increased when free text was included. For example, the prevalence of chest pain within 7 days prior to MI would be underestimated by a third if free text were ignored (18.9% instead of 27.2%) (Table 2).

**Table 2:** Information available in CPRD primary care data (coded data and free text) for a random sample of 2000 patients with myocardial infarction in the linked CALIBER dataset

Data element	Structured data only		Structured or free text		% increase by using free text
Within 90 days before or after MI:					
Pulse rate	323	(16.2%)	634	(31.7%)	96%
Blood pressure	1557	(77.9%)	1609	(80.5%)	3%
Left ventricular function result	115	(5.8%)	309	(15.5%)	169%
Coronary angiogram results	26	(1.3%)	198	(9.9%)	662%
Irregular pulse	2	(0.1%)	6	(0.3%)	200%
Atrial fibrillation or flutter	121	(6.0%)	153	(7.6%)	26%
Chest pain ≤7 days before MI	378	(18.9%)	543	(27.2%)	44%
Chest pain ≤90 days before MI	455	(22.8%)	642	(32.1%)	41%
Shortness of breath ≤7 days before MI	62	(3.1%)	102	(5.1%)	65%
Shortness of breath ≤90 days before MI	125	(6.3%)	196	(9.8%)	57%

### Classifying the type of myocardial infarction

Among the 608 patients with a MINAP record giving detailed information about the MI, 149 (25%) had a Read code stating the type of MI and 46 (8%) had this information available only in the free text. Inclusion of free text information increased the sensitivity for detection of MI subtypes, with a slight reduction in sensitivity and positive predictive value (Table 3). Concordance of derived MI subtypes with the MINAP gold standard was lower for free text (78.3%; 95% CI 63.6%, 89.1%) than Read codes (91.9, 95% CI 86.4%, 95.8%).

**Table 3:** Type of MI as recorded in CPRD primary care data, for patients with a ‘gold standard’ MI subtype record in MINAP

		Subtype of MI	
Primary care source of type of MI		STEMI (*N* = 315)	NSTEMI (*N* = 293)
Structured (Read codes) (number of patients)	STEMI	41	6
	NSTEMI	6	96
Free text (number of patients)	STEMI	13	5
	NSTEMI	5	23
Patients with no information on type of MI in primary care		250	163
Accuracy of MI classification using structured data	Sensitivity, %	13.0 (9.5, 17.2)	32.8 (27.4, 38.5)
	Specificity, %	98.0 (95.6, 99.2)	98.1 (95.9, 99.3)
	Positive predictive value, %	87.2 (74.3, 95.2)	94.1 (87.6, 97.8)
Accuracy of MI classification using structured and free text data	Sensitivity, %	17.1 (13.1, 21.8)	40.6 (34.9, 46.5)
	Specificity, %	96.2 (93.4, 98.1)	96.5 (93.8, 98.2)
	Positive predictive value, %	83.1 (71.7, 91.2)	91.5 (85.4, 95.7)

### Validity of myocardial infarction records

Our manual review of coded and free text data concluded that the majority of patients with MI recorded only in CPRD primary care data (210/267) had a genuine recent MI. In some cases, free text contributed directly to our decision; for example, one patient had an MI code on the same date as a mental health diagnosis, but the free text stated that the MI was one year ago. We assessed whether information in the free text could improve the performance of machine learning models for identifying a true MI. Using the Random Forest model on the entire sample of 2000 patients, the percentage correct (mean and 95% bootstrap confidence interval) without using free text was 95.9% (95% CI 94.3%, 97.4%) and using free text was 95.6% (95% CI 93.9%, 97.3%); i.e. no significant difference.

### Cause of death

Cause of death was incompletely recorded in CPRD primary care data, with only a slight improvement over time. Free text contributed 37% of the causes recorded in primary care (381/1022) (Table 4). Only 46.7% of patients (95% CI 43.6%, 49.8%) had the same exact ICD-10 code for the underlying cause in primary care and the death registry, but in 72.5% (95% CI 69.7%, 75.2%) the cause was from the same ICD-10 chapter. CPRD primary care data had high specificity but moderate sensitivity for identifying coronary, cerebrovascular or cancer deaths, but no difference in accuracy between structured and free text records (Table 5).

**Table 4:** Proportion of deaths with a cause recorded in CPRD primary care data (*N* = 600 for each 3-year band)

How cause of death is recorded in primary care	Years 2001–2003	Years 2004–2006	Years 2007–2009	Accuracy (95% CI)
Transcribed death certificate entry (e.g. 1a Heart failure, 1b Acute myocardial infarction)				
Read codes	46 (7.7%)	103 (17.2%)	112 (18.7%)	59% (52%, 65%)
Free text	32 (5.3%)	41 (6.8%)	47 (7.8%)	53% (44%, 63%)
Explicit cause of death (e.g. Cause of death: myocardial infarction)				
Read codes	26 (4.3%)	47 (7.8%)	36 (6.0%)	30% (22%, 40%)
Free text	16 (2.7%)	17 (2.8%)	16 (2.7%)	55% (40%, 69%)
Cause of death implied by diagnosis dated on or after date of death				
Read codes	140 (23.3%)	79 (13.2%)	52 (8.7%)	44% (37%, 51%)
Free text	69 (11.5%)	67 (11.2%)	76 (12.7%)	40% (34%, 46%)
No cause of death in CPRD	271 (45.2%)	246 (41.0%)	261 (43.5%)	–

**Table 5:** Accuracy of underlying cause of death in CPRD primary care data compared to the death registry gold standard, for the 1022 individuals with cause of death recorded in both sources. For coronary deaths not recorded as coronary in CPRD, the most common causes in CPRD were I469 ‘Cardiac arrest’, I500 ‘Congestive heart failure’ and I501 ‘Left ventricular failure’. For stroke deaths not recorded as stroke in CPRD, the most common causes in CPRD were ‘J180 Bronchopneumonia, unspecified’, ‘J189 Pneumonia, unspecified’ and ‘F03X Unspecified dementia’

Source of cause of death record in CPRD	Free text	Coded
Number of deaths	381	641
Same underlying cause	184 (48.3%)	293 (45.7%)
Same 2-character ICD-10 code for underlying cause	222 (58.3%)	371 (57.9%)
Same ICD-10 chapter for underlying cause	278 (73.0%)	463 (72.2%)
Coronary deaths (ICD-10 I20–I25, *N* = 163):		
Sensitivity, %	65.3 (50.4, 78.3)	68.4 (59.1, 76.8)
Specificity, %	97.9 (95.7, 99.1)	98.3 (96.8, 99.2)
Cerebrovascular deaths (ICD-10 F01, I60–I69, *N* = 101):		
Sensitivity, %	66.7 (51.6, 79.6)	58.5 (44.1, 71.9)
Specificity, %	98.5 (96.5, 99.5)	97.8 (96.2, 98.8)
Cancer deaths (ICD-10 C00–C97, *N* = 268):		
Sensitivity, %	93.0 (86.1, 97.1)	80.4 (73.5, 86.1)
Specificity, %	95.7 (92.7, 97.8)	98.5 (97.0, 99.4)

## Discussion

We analysed a larger quantity of unstructured free text in a UK primary care database than any previous study. We were able to do so by using natural language processing software to extract information without requiring manual review or anonymisation of the free text record. Free text notes in primary care records commonly contain brief expressions, non-grammatical phrases, spelling mistakes and irregular punctuation, posing a particular challenge to NLP tools [17]. There have been attempts to better phenotype myocardial infarctions using NLP on hospital discharge summaries [18], but none using primary care data. Overall there have been very few NLP studies on free text in UK primary care [7–10]; this is the largest to date. Under CPRD policy at the time of the study, free text required manual anonymisation by CPRD staff before being released to researchers, and anonymising the 705,523 words analysed in this project would have cost over £35,000. We previously carried out a validation study of myocardial infarction (MI) in the CALIBER linked EHR resource. We found that agreement between the data sources in CALIBER was poor [12]. MI records in primary care data typically did not differentiate between subtypes of MI (STEMI, ST segment elevation MI, or NSTEMI, non ST segment elevation MI), despite the clinical importance of this distinction.

### Summary of main findings

We found that free text contained a large amount of information on symptoms, test results (e.g. left ventricular function), clinical measurements, diagnoses, admissions and administration (e.g. sickness certificates), much of which was not present in the structured data, and could potentially be useful for clinical research studies. Free text contained a large number of records of suspected conditions, for which the clinical system does not provide a facility for structured recording. Symptoms related to myocardial infarction such as chest pain and shortness of breath were recorded in the free text rather than as Read codes in about a third of patients, because the information was scanned as an image and not converted to text.

We attempted to use free text to help determine if a MI record in CPRD with no linked MI record in another data source was a true current MI, rather than an incorrectly dated historic event. Based on manual review of the FMA-annotated CPRD record, we concluded that the vast majority were true current MI, which limited our ability to quantify the contribution of the free text for such determination. The small number of incorrect MI records made this a difficult machine learning task, as it is known that imbalance in datasets for machine learning can lead to a biased classifier. Potential methods of improving performance on such tasks may be to alter the training balance [19], or develop bias-aware probabilistic classifiers [20].

Cause of death was incompletely recorded, even with the addition of free text, and in a significant proportion of cases the cause of death in the death registry and in primary care were different. This may be because the general practitioner did not receive definitive cause of death information from post mortems or coroner reports; cause of death information was more complete and accurate for cancer deaths, where there is less ambiguity. Linked registry data seems to be the only complete and accurate source of cause of death data.

### Limitations

Research studies incorporating NLP must include validation of variables derived using NLP, which is usually done by manual review of a random subset of the records. The main limitation of our study was that we were unable to manually validate the extracted data items against the original raw text, because CPRD withdrew access to free text for researchers part-way through the study (and no longer collects free text). We refer to a previous validation of the Freetext Matching Algorithm demonstrating over 90% precision [7], which is adequate for this project demonstrating the broad utility of free text in primary care, but studies with clinical implications would require the NLP error rate for specific variables to be propagated into the uncertainty of the final estimates. For some measures we were able to compare information extracted from text with linked registry datasets (MINAP and the death registry).

Another limitation is that we used only one natural language processing algorithm; other open source annotators have been released since the development of the FMA. Examples include cTakes [21] (Mayo Clinic), MetaMap [22] (US National Library of Medicine), Hitex [23] (Harvard Medical School) and Bio-Yodie, developed as part of the KConnect Horizon 2020 project [24]. For this project we used our in-house FMA algorithm because of its small size and simplicity. We limited the sample size in order to be able to solve any unexpected problems, and to facilitate CPRD’s process of assuring that the output contained only numerical data, with no unintended ‘leak’ of text.

Another limitation was the use of only a single annotator for the cause of death classification. The ICD-10 rules for selecting the underlying cause of death are complex; in this study this task was performed by a clinician with experience in classifying the cause of death for over 2000 patients in a previous study [7]. It would have required considerable resource to train another annotator to the same level. Given that agreement between the primary care record and death registry was poor even with a well-trained annotator, it was unlikely that additional annotation would alter the conclusion that the primary care record is an unreliable source of cause of death information.

### Clinical implications

Although there has been much research activity around natural language processing of clinical text, few advances have made it to the clinic [25]. A fundamental problem is that information extracted from text cannot be relied upon to be completely accurate because of the nuances of human language; an error rate of 5% may be accommodated in research but is not an acceptable risk when planning treatment for an individual patient. A potential solution is to embed real-time natural language processing within clinical systems, to generate structured data whilst giving clinicians the freedom to express their thoughts in a natural way. The NHS Common User Interface guidelines [26] contains recommendations for such technology, but current systems have not yet implemented it in practice.

### Research implications

NLP has been applied to primary care records in other countries for research studies attempting early diagnosis of multiple sclerosis [27], classification of childhood respiratory illnesses [28, 29] and identification of heart failure symptoms [30]. However, NLP of primary care notes can be challenging – the language is terse, often ungrammatical and abbreviated [17]. The sublanguage of primary care clinical notes has not been studied at scale, nor are we aware of international comparisons in this area, which would be helpful for generalising NLP methodology worldwide.

One of the difficulties in healthcare text analytic research is the governance and access restrictions on the use of free text. In the UK, CPRD no longer provides access to primary care free text, following the Information Commissioner’s Office instructions (https://ico.org.uk/), leaving The Health Improvement Network as the only UK primary care research database containing free text. CPRD can facilitate GP questionnaires to validate or enhance a small sample of records (at additional cost), but large-scale research using CPRD will be based entirely on the coded data. In the long term, incentives such as the Quality and Outcomes Framework [31] may help to improve data completeness for specific data items that are clinically important.

However, in some secondary care NHS Trusts, clinical text is available for research under secure governance arrangements. The South London and Maudsley NHS Trust has been using the Cogstack architecture [32] to analyse clinical text for mental health research for a number of years [33]. At Kings College Hospital, a similar system is in use for audit and quality improvement, and is undergoing ethical review for use for research. The value of primary care free text as demonstrated in our study and others [8, 9] makes the case for investment in systems to enable natural language processing on primary care free text at source, with appropriate governance to maximise the clinical benefits of such research whilst protecting the confidentiality of patient data.

## Conclusion

Unstructured free text in primary care records contains much information that is potentially useful for research and is not recorded in the structured data, such as symptoms, investigation results and specific diagnoses. Natural language processing to convert this information into a structured form can enrich primary care data at scale for research, and potentially yield population-based insights into early presentations of disease.

## Data Availability

The data that support the findings of this study are available from CPRD, but restrictions apply to the availability of these data, which were used under license for the current study, and so are not publicly available. This project uses the CALIBER dataset, which is de-identified (pseudonymised) but is sufficiently detailed to be considered sensitive data with a potential risk of patient re-identification if combined with other data sources, and the terms of the data sharing agreement do not permit it to be shared. Access to the database for research can be obtained by submitting an application to the CPRD Independent Scientific Advisory Committee. All the software used in this project is available as open source software. The Freetext Matching Algorithm is available on Github: https://github.com/anoopshah/freetext-matching-algorithm and the lookups are on https://github.com/anoopshah/freetext-matching-algorithm-lookups. Operating system: Windows, or Linux with wine and Visual Basic 6 runtime. Programming language: Visual Basic 6. License: GNU General Public License v3.0.

## Abbreviations

CALIBER: Clinical Research using Linked Bespoke datasets and Electronic Records
CPRD: Clinical Practice Research Datalink
EHR: electronic health record
FMA: Freetext Matching Algorithm
GP: general practitioner
HES: Hospital Episode Statistics
ICD: International Classification of Diseases
MI: myocardial infarction
MINAP: Myocardial Ischaemia National Audit Project
NHS: National Health Service
NLP: natural language processing
NSTEMI: non ST elevation myocardial infarction
ONS: Office for National Statistics
STEMI: ST elevation myocardial infarction
UK: United Kingdom
